# Misuse of Topical Corticosteroids for Cosmetic Purpose in Antananarivo, Madagascar

**DOI:** 10.1155/2017/9637083

**Published:** 2017-08-21

**Authors:** F. A. Sendrasoa, I. M. Ranaivo, M. Andrianarison, O. Raharolahy, N. H. Razanakoto, L. S. Ramarozatovo, F. Rapelanoro Rabenja

**Affiliations:** Department of Dermatology, University Hospital Joseph Raseta Befelatanana, Antananarivo, Madagascar

## Abstract

This cross-sectional study was conducted in Antananarivo, Madagascar, from June to September 2012. We aim to evaluate the misuse of TC on the face for cosmetic purpose and the adverse effects due to its application. A questionnaire-based analysis was done among females who use topical corticosteroids on the face for cosmetic purpose. Of the 770 women questioned, 384 (49,8%) used topical corticosteroids for cosmetic purpose whose mean age was 38 years (range 16–73 years). Two hundred and sixty-one females (68%) used TC combined with handcrafted cosmetics, and 123 (32%) used TC alone. “Pandalao,” which contains salicylic acid, peppermint oil, lanolin, powder of Juanes de Vigo (mercury powder), and Vaseline, is the most handcrafted cosmetic combined with TC in our study (used by 29,4% respondents). Only one (0,26%) had obtained the TC by physician's prescription, 234 (61%) from cosmetic retailers, 92 (23%) directly from local pharmacies, 49 (12%) from beauticians, and 15 (4%) from unspecified sources. Lightening of skin color was the main reason for using TC in 44,8% of respondents in the absence of any primary dermatosis. Pigmentation disorders (63,2%) and cutaneous atrophy (52,1%) were the most adverse effects noted.

## 1. Introduction

Topical corticosteroids (TC) have been used for more than six decades to treat various dermatological disorders due to their wide range of action [1, 2]. As several countries where potent TC are easily available over-the-counter at a low price, misuse has been noticed among the general population, producing many adverse effects [3, 4]. To our knowledge, in spite of being a common problem, no study has investigated the misuse of topical corticosteroid products in Madagascar. This study was conducted among females living in Antananarivo, Madagascar, to assess the frequency of TC misuse, the reasons behind it, and the most common adverse effects resulting from it.

## 2. Methodology

A cross-sectional study was conducted from June to September 2012 in Antananarivo which is the capital of Madagascar. Investigations were done in municipal markets, popular neighborhoods in Antananarivo, pharmacies, beauty salons, and supermarkets.

A questionnaire-based analysis was done among females who use topical corticosteroids alone or associated with other products, on the face for cosmetic purpose. Of the 770 women questioned, 384 respondents were included. The following data were collected: characteristics of study participants (age, educational level), class of topical corticosteroids, reasons of use, adverse effects, and conditions of issue. A full skin examination was performed to detect any condition related to abuse of TC. Most of the diagnoses were exclusively clinical and were based on the typical, classical features.

Patient's attitudes toward the side effects were also evaluated in our study.

*Statistical Analyses*. Data was analysed by “R” software created by Robert Gentleman and Ross Ihaka, in the Department of Statistics at the University of Auckland in New Zealand.

## 3. Results

One hundred and thirty-seven females (35,6%) in the age group of 35–44 years used TC (Table 1) and 34,4% were illiterate.

Of the total 384 respondents, 261 (68%) used TC combined with handcrafted cosmetics, and 123 (32%) used TC alone. All females enrolled into study had a history of using topical corticosteroids for at least 3 weeks. The various classes of TC used by patients and the duration use were detailed in Table 2. “Pandalao” is the most handcrafted cosmetic combined with TC in our study (used by 29,4% respondents), which contains salicylic acid, peppermint oil, lanolin, powder of Juanes de Vigo (mercury powder), and Vaseline.

Of the 384 respondents, only one (0,26%) had obtained the TC on physician's prescription, 234 (61%) from cosmetic retailers, 92 (23%) directly from local pharmacies, 49 (12%) from beauticians, and 15 (4%) from unspecified sources.

One hundred and twenty-two respondents prepared themselves compositions containing topical corticosteroids, 52% used 15 g of TC per month, 23% used 22,5 g, and 5% used 30 g per month.

Lightening of skin color was the main reason for using TC in 44,8% of respondents in the absence of any primary dermatosis.

**Figure 1:**
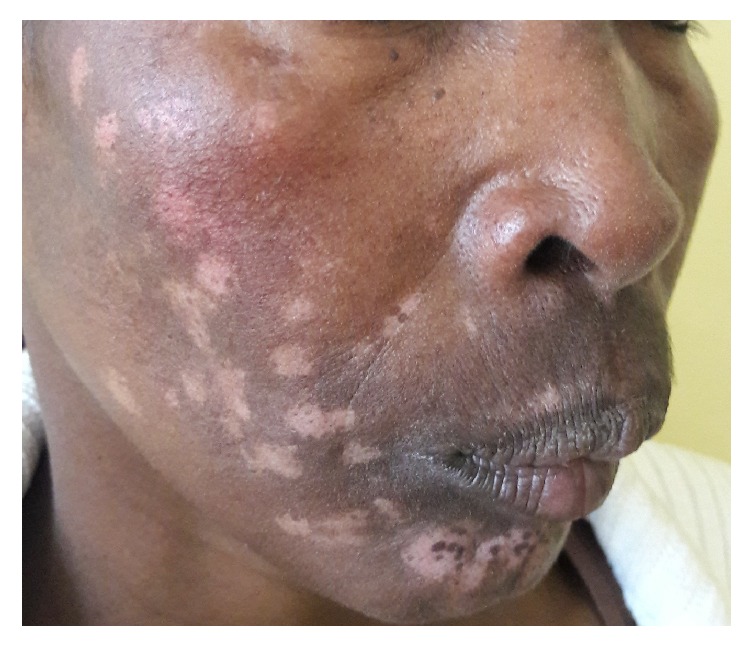
Steroid induced hypopigmentation.

**Figure 2:**
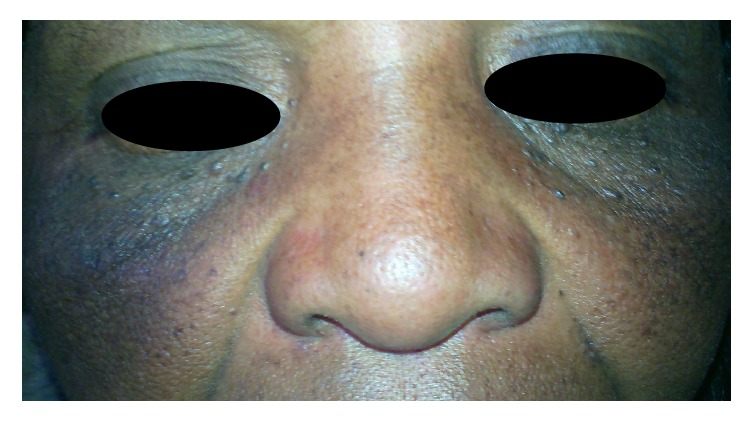
Steroid induced hyperpigmentation.

**Figure 3:**
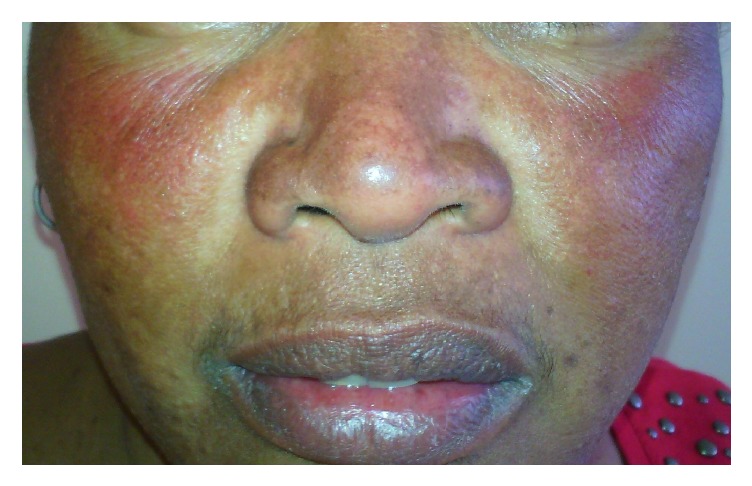
Erythema induced by topical corticosteroids.

**Figure 4:**
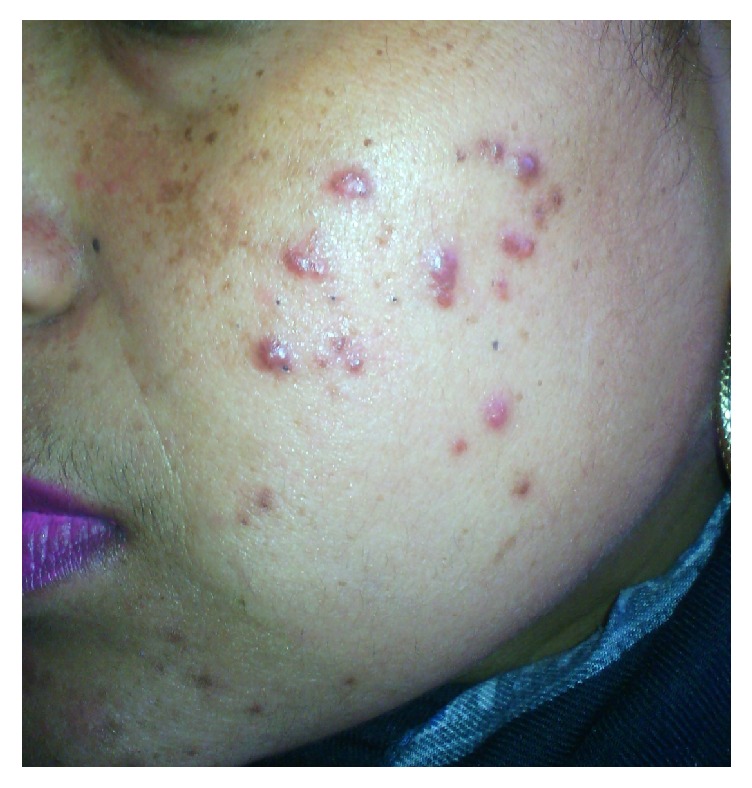
Steroid acne.

Only 5 (1,3%) respondents did not present dermatological adverse effects of TC. The dermatological adverse effects of TC are listed in Table 3. Pigmentation disorders and cutaneous atrophy were the most adverse effects noted. Other adverse effects were steroid induced erythema, steroid dependency, hirsutism, and acne. Photographs representing some of the adverse effects are shown in Figures 1–4.

Only 13% of topical corticosteroids users intended to seek for dermatological care following cutaneous side effects. The cost of dermatological care was not affordable according to 152 respondents (45,2%) and 84 (25%) were convinced of the product's effectiveness.

## 4. Discussion

Our study shows that Malagasy women do not hesitate to use topical corticosteroids on the face for cosmetic purpose. Malagasy purchasing power was limited to buy luxury cosmetics. This may explain the misuse of topical corticosteroids which is affordable. Potent topical corticosteroids were the most used. We conducted our study only in women because facial care is not yet daily habits for Malagasy men.

The first TC was introduced to the world of dermatology in 1952 by Sulzberger and Witten [1]. TC are principally used for their anti-inflammatory, antiproliferative, and immunosuppressive properties [2]. These properties confer upon them the ability to cure a wide variety of disorders.

Misuse of TC appears to be a common problem, as reported by many studies in India, China, Iraq, and Senegal [3–9]. Like these countries [10], in Madagascar, most TC are available at throwaway prices since they come under drug control price order and they are mostly sold as over-the-counter products. This gives rise to multiple problems. However, most developed countries restrict the sales of TC strictly by prescription, because they should be used judiciously, for appropriate indications and duration. As in many African countries, Malagasy women face pressure to lighten their skin due to the widespread social perception that light skin is considered more attractive and reflective of high social status [11, 12].

The facial skin is vulnerable by TC because the facial skin is thinner than the skin of most other parts of the body. This results in increased percutaneous absorption of drugs. The sebaceous glands on the face are larger than elsewhere, and there is an increased tendency to sweating particularly in tempered and humid climates as is prevalent in most parts of Madagascar. Hence, it is more liable to the ill-effects of environmental factors such as sunlight and pollution, friction due to cleaning and rubbing, and application of cosmetics and drugs such as TC.

As in Accra Ghana [13], pigmentation disorders were the commonest adverse effects in our study. People with types IV to VI (like Malagasy people) are particularly affected by hypopigmentation; however, it is not noticed frequently in very light skinned individuals [14]. This difference may be related also to compositions used. In India where Panderm Plus Cream and Kligman's formula were widespread, the commonest adverse effect was acne [7, 15, 16]. In China, Lu et al. show that dermatitis and acne were the widest adverse effects [4].

Cutaneous atrophy after TC use is quite common; it was the second adverse effect observed in our result. Atrophic changes can affect both epidermis and dermis. Microscopic degenerative changes in epidermis are evident following 3–14 days of treatment. TC induce resorption of mucopolysaccharide ground substance in the dermis. Repeated use in the same area causes epidermal thinning and changes in connective tissue of dermis leading to lax, transparent, wrinkled, and shiny skin along with striae, fragility, and prominence of underlying veins. The loss of connective tissue support for dermal vasculature results in erythema, telangiectasia, and purpura [17]. Degree of skin atrophy is influenced by age, body site, potency, and presence of occlusion. Furthermore, systemic adverse effects from TC, which were not evaluated in our study, have also been described and they are more likely to develop when highly potent TC are used for prolonged periods on thin skin like facial skin or on raw surfaces [17, 18].

“Pandalao” which contains powder of Juanes de Vigo (mercury powder) is the most handcrafted cosmetic combined with TC in our study. The toxicity of mercury has been well known; the main adverse effect of the inorganic mercury is kidney damage [19]. Mercury may also cause skin rashes, skin discoloration, and scarring, as well as a reduction in the skin's resistance to bacterial and fungal infections [3, 20]. Distribution of mercury containing creams and soaps should be banned in Madagascar like in European Union and numerous African nations [21, 22].

## 5. Conclusion

Our study revealed that misuse of topical corticosteroids is an important public health problem where several sectors were involved. TC are sold in cosmetic retailers and do not need prescription in pharmacies. The over-the-counter use of TC has a psychological impact on the user; no restriction use of TC ensures users about the safety of this product. In addition, misuse of TC shows that the supply channel of this product is defective and illegal in Madagascar. A task force against TC abuse should be formed in Madagascar, which seeks to raise public awareness, run media campaigns, form study for doctors, highlight the problem in journals, and meet with central and state authorities.

## Figures and Tables

**Table 1:** Total number of respondents' age group and educational level.

	Number of patients (%)	*p* ^*∗*^
Age group (years)		
15–24	21 (5,4)	
25–34	83 (21,6)	
35–44	137 (35,6)	<0,05^*∗*^
45–54	101 (26,3)	
≥55	42 (10,9)	
Educational level		
Illiterate	132 (34,4)	<0,05^*∗*^
Primary school	97 (25,2)	
Secondary school	81 (21)	
High school	46 (12)	
University	28 (7,3)	

^*∗*^The use of TC is common in the age group of 35–44 years and illiterate females.

**Table 2:** Various classes of TC used by patients and duration use.

	Number of patients (%)	*p* ^*∗*^
*Various classes*		
Class I (superpotent)	4 (1)	
Class II (potent)	96 (25)	
Class III (least potent)	23 (6)	
Handcrafted cosmetic containing TC	261 (68)	
*Duration use*		
3 weeks–3 months	41 (10,6)	
3 months–11 months	50 (13)	
1–5 years	184 (48)	<0,05^*∗*^
6–10 years	47 (12,2)	
>10 years	62 (16,1)	

^*∗*^Common duration use of TC is between 1 and 5 years.

**Table 3:** Dermatological adverse effects.

	Number of patients (%)	*p* ^*∗*^
None	5 (1,3)	
Pigmentation disorders	243 (63,2)	<0,05^*∗*^
Cutaneous atrophy	204 (53,1)	
Steroid induced erythema	202 (52,6)	
Steroid dependency	161 (41,9)	
Hirsutism	80 (20,8)	
Contact dermatitis	76 (19,8)	
Acne	66 (17,1)	
Stretch marks	19 (5)	
Other adverse effects	12 (3,1)	

^*∗*^Pigmentation disorders (63,2%) were the most adverse effects noted.
